# Perineural Invasion (PNI) Definition, Histopathological Parameters of PNI in Oral Squamous Cell Carcinoma With Molecular Insight and Prognostic Significance

**DOI:** 10.7759/cureus.40165

**Published:** 2023-06-09

**Authors:** Parth R Goswami, Gyanendra Singh

**Affiliations:** 1 Pathology, All India Institute of Medical Sciences, Rajkot, Rajkot, IND

**Keywords:** pni histopathology, squamous cell carcinoma, survival rate, oral scc, pni

## Abstract

Oral squamous cell carcinoma is associated with severe morbidity, recurrence of tumor, and reduced survival rate despite advances in treatment. Perineural invasion (PNI) is associated with neurotropic malignancy. PNI is due to the tropism of cancer cells toward nerve bundles in tissue. The aim of this literature review is to study the definition, patterns of PNI, Prognostic and therapeutic significance, and mechanism of PNI along with a molecular insight into oral cavity squamous cell carcinoma. Liebig type A pattern defines PNI as the presence of tumor cells within the peripheral nerve sheath & infiltration into the epineurium, perineurium, or endoneurium. Liebig type B pattern defines PNI as a tumor encircling at least 33% of a nerve. Few studies demonstrated an association between PNI and cervical metastasis which indicate poor prognosis. A higher level of expression of nerve growth factor and tyrosine kinase is associated with PNI in OSCC which can be considered as a biomarker of PNI. PNI needs to be studied in detail as it is associated with the aggressiveness of the tumor and decreased survival.

## Introduction and background

Oral cavity cancer is the sixth most prevalent cancer worldwide, with squamous cell carcinoma accounting for 90% of cases [1,2]. Despite advances in treatment, oral squamous cell carcinoma is associated with high morbidity, tumor recurrence, and a low-rate survival rate [3].

Perineural invasion (PNI) is a neurotropic malignancy associated with aggressive behavior, increasing morbidity and mortality with decreased five-year survival [4]. Moreover, numerous clinicopathological factors, such as tumor stage, grade, depth of invasion, lymph node metastasis, and tumor buds, among others, influence the prognosis of oral squamous cell carcinoma.

Furthermore, PNI incidence in head and neck cancer is around 80% [5]. It is associated with neurotropic malignancy. PNI is caused by the tendency of cancer cells to migrate toward nerve bundles in the tissue. Although PNI is a form of metastasis, it is distinct from lymphovascular invasion. In PNI, tumor cell travel along the nerve pathways, causing pain and distal spread of the tumor [6,7]. In addition, PNI is a known independent predictor for colorectal carcinoma and salivary gland malignancy [8,9]. This literature review aims to study the definition, patterns of PNI, prognostic and therapeutic significance, mechanism of PNI, and molecular insight into oral cavity squamous cell carcinoma.

## Review

PNI definition

PNI was first described as head and neck cancer by Cruveiheir in 1835. To the best of our knowledge, different literature reviews define PNI differently. Thus, no standard definition exists among pathologists. Herewith, we will review every definition of PNI from various studies.

In 1985, Batsakis broadly defined PNI as tumor cell invasion in, around, and through the nerves. Dunn et al. define PNI as the presence of malignant squamous cells in perineural space along with total or near total circumference involvement of nerves in the histopathology section [10].

Liebig et al. proposed the most accepted definition of PNI as two histopathological patterns. Liebig type A pattern defines PNI as the presence of tumor cells within peripheral nerve sheaths and infiltration into the epineurium, perineurium or endoneurium.

Liebig type B pattern defines PNI as a tumor encircling at least 33% of a nerve. However, this definition does not distinguish between perineural spread (detection of cells in and around the perineural space) and intraneural spread (cell penetration within the nerves), which can affect prognosis. Thus, further study is required [4,11].

**Figure 1:**
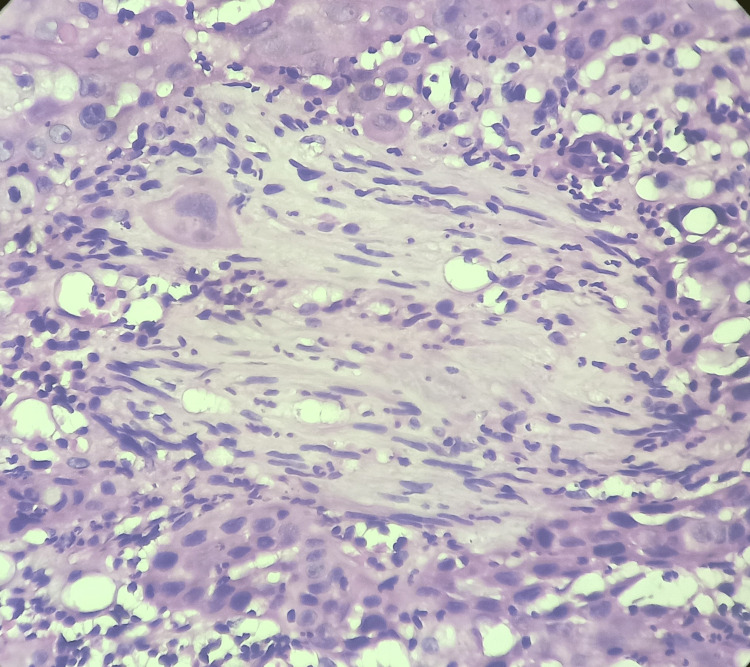
Microphotograph of PNI in oral squamous cell carcinoma

In addition, Versha et al. [12] considered PNI positive for detecting tumor cells in the perineural space or epineurium. Figure 1 shows a histopathological image of PNI.

PNI positivity in oral cavity squamous cell carcinoma

In the Versha et al.'s study, 40.5% of squamous cell carcinoma patients showed PNI. The findings of PNI positivity in squamous cell carcinoma in various studies are summarized in Table 1 [13-16].

**Table 1:** PNI positivity in oral squamous cell carcinoma

Serial Number	Reference Number	Percentage of Positivity in Primary Oral Squamous Cell Carcinoma
1.	12	40.5%
2.	13	22.03%
3.	14	40.0%
4.	15	17%
5.	16	2.5-71%

According to Kurtz et al. [17], there is a marked variation in PNI frequency in squamous cell carcinoma, ranging from 2% to 82%. This variation could be due to a lack of standard definitions and detection methods.

PNI and node involvement relationship

As previously discussed, PNI is likely to be associated with aggressiveness, poor survival and metastasis to lymph nodes due to invasion of perineural space. Few studies have found an association between PNI and cervical metastasis indicating poor prognosis [18-20]. Table 2 shows the relationship between PNI and the T3, T4 TNM stages.

**Table 2:** PNI positivity in T3 or T4 stage in primary oral squamous cell carcinoma

Serial Number	Reference Number	PNI Positivity in T3 or T4 Stage in Primary Oral Squamous cell Carcinoma
1.	12	40%
2.	13	80%
3.	15	70.5%

Zubair et al. [13] showed a statistically significant correlation between PNI and lymph node involvement using the chi square test. The authors found that PNI was present in 49.3% of clinically positive nodes. Hence, as a result of the correlation between PNI and nodal metastasis, it can be concluded that PNI could be an indicator of elective neck dissection or adjuvant therapy [14].

Mechanism and histopathological reporting of PNI

PNI was previously thought to be caused by the passive mechanical extension of cells along less resistant connective tissue of the perineurium or epineurium lymphatics. However, this theory has not been accepted due to the advancement of ultrastructural scans of nerve sheaths, which shows resistant barriers that separate nerve from and surrounding tissues [21].

Bockman concluded that tumor cells do not invade nerve passively; instead, they penetrate the perineurium by association with Schwann cells and axons in the endoneurium [22]. The current review will discuss the molecular insights of PNI in squamous cell carcinoma to understand whether it could be a potential target for advanced treatment of squamous cell carcinoma.

Three histopathological parameters can be reported when PNI is present.

PNI Location

Miller et al. [14] provided criteria for estimating PNI locations. For this reason, the distance to the tumour’s edge can be measured in millimetres at each focal point. If the value is positive, it is considered an extra tumoral location. If the value is negative, it is considered an intratumoral location.

If the focus of PNI is 0-0.2 mm from the tumor edge, it can be randomly considered a peripheral location.

PNI Density

It is calculated by counting the number of PNI foci per tissue per section. Additionally, it can be categorized as 1-3 nerves per section or >3 nerves per section. According to Versha et al., 80% of PNI positive cases showed a PNI density of 1-3 nerves per section. Thus, more studies should be conducted to check the association of PNI density with prognosis.

PNI Pattern

Different patterns of nerve involvement include complete encirclement, incomplete “crescent” like encirclement, sandwiching onion skin, partial invasion and neural permeation. Rahima et al. identified complete and incomplete encirclement as the most prevalent pattern of PNI.

Further, Versha et al. identified incomplete or crescent like encirclement as the most prevalent pattern of PNI. Reporting protocols published by the Royal College of Pathologists in the UK and the College of American Pathologists guidelines include the presence or absence of PNI invasion in oral squamous cell carcinoma [23]. Thus, it is recommended that rather than reporting the presence or absence of PNI, the quantification and other parameters of PNI should be studied in detail [24].

PNI and recurrence survival rate relation

The TNM staging and numerous histopathological factors, such as tumor buds, lymphoplasmacytic inflammation, lymphovascular invasion, etc. determine the prognosis of oral squamous cell carcinoma. When assessing the prognosis of oral squamous cell carcinoma, PNI is one of the factors to consider. In studies of oral squamous cell carcinoma, many researchers have found that PNI is linked to disease recurrence, increased risk of metastasis, and a reduced five-year survival rate [4,11,25,26].

In a number of other studies, PNI in oral squamous cell carcinoma associated with disease recurrence increased the risk of regional and distant metastasis as well as poor survival rates [27-30].

PNI and nerve size, depth of invasion

Rahima et al. [29] concluded that the depth of invasion and PNI strongly correlate. Increasing tumour thickness increases the like hood of cells penetrating nerve fibres and the possibility of poor prognosis.

Aivazian et al. [31] concluded that PNI in larger nerves that are greater than 1 mm in diameter was significantly associated with local recurrence. Woolgar et al. [27] also concluded that PNI in major nerves or nerves with small sizes (<1 mm) was associated with reduced survival rates and increased recurrence of squamous cell carcinoma. However, Zubair et al [13] found no association between nerve size and the recurrence of squamous cell carcinoma. Therefore, additional research is required to comprehend the association between PNI and nerve size.

PNI and molecular insight

The activation of three signalling pathway, including neurotropic factors, extracellular matrix adhesion protein, and chemotaxis regulators, is probably necessary for PNI by cancer cells and migration towards nerves.

Mclaughlin et al. [32] found a positive correlation between N CAM neural cell adhesion molecule expression and PNI. Other adhesion molecules like ICAM -5, Claudin 1 and Claudin 4 expression are also associated with PNI [33,34].

In oral squamous cell carcinoma, the extracellular matrix protein laminin 5 is also correlated with elevated PNI [35]. However, the most accepted molecular mechanism of PNI is the overexpression of NGF (nerve growth factor) and TrKA.

Few studies demonstrated a strong association between NGF and TrKA and PNI in oral squamous cell carcinoma [36-38]. It was also found that PNI is associated with pain [15]. Zuda et al. [15] found a significant correlation between NGF and TrKA overexpression and lymphovascular invasion and an increased depth of invasion >4 mm in oral squamous cell carcinoma, indicating that NGF & TrkA are associated with aggressiveness of tumour. Thus, understanding microenvironment and molecular insight of PNI could be potential target for treating PNI associated with squamous cell carcinoma for a better prognosis. Table 3 shows the prognostic impact of PNI in oral squamous cell carcinoma.

**Table 3:** Prognostic impact of PNI in oral squamous cell carcinoma

Serial Number	Reference Number	Prognostic Impact of PNI in Oral squamous cell carcinoma
1.	12,4,11	PNI is associated with recurrent disease, an increased probability of metastasis, and decreased five-year survival
2.	30	PNI is associated with the recurrence of squamous cell carcinoma and is predictive of extracapsular spread
3.	24	PNI predicts worse disease-free survival
4.	13	Significant association found between PNI occurrence, nodal metastasis and recurrence
5.	23	PNI is a pathological parameter to predict local relapse, node metastasis and poor survival outcome

## Conclusions

In oral squamous cell carcinoma, PNI is associated with disease recurrence, an increased risk of metastasis, and a decreased five-year survival rate. In order to gain a comprehensive understanding of PNI, we report its various definitions, association with survival and nodal metastasis, histopathological parameters, mechanism, and molecular insight. As a biomarker of PNI, increased expression of nerve growth factor and tyrosine kinase has been linked to PNI in OSCC. Targeted oral squamous cell carcinoma therapy can be planned with a deeper understanding of PNI and molecular insight.
